# Water Microbiology. Bacterial Pathogens and Water

**DOI:** 10.3390/ijerph7103657

**Published:** 2010-10-15

**Authors:** João P. S. Cabral

**Affiliations:** Center for Interdisciplinary Marine and Environmental Research (C. I. I. M. A. R.), Faculty of Sciences, Oporto University, Rua do Campo Alegre, 4169-007 Oporto, Portugal; E-Mail: jpcabral@fc.up.pt; Tel.: +351-220402751; Fax: +351-220402799

**Keywords:** drinking water, cholera, typhoid fever, bacillary dysentery, fecal indicator bacteria, coliforms, ammonia

## Abstract

Water is essential to life, but many people do not have access to clean and safe drinking water and many die of waterborne bacterial infections. In this review a general characterization of the most important bacterial diseases transmitted through water—cholera, typhoid fever and bacillary dysentery—is presented, focusing on the biology and ecology of the causal agents and on the diseases’ characteristics and their life cycles in the environment. The importance of pathogenic *Escherichia coli* strains and emerging pathogens in drinking water-transmitted diseases is also briefly discussed. Microbiological water analysis is mainly based on the concept of fecal indicator bacteria. The main bacteria present in human and animal feces (focusing on their behavior in their hosts and in the environment) and the most important fecal indicator bacteria are presented and discussed (focusing on the advantages and limitations of their use as markers). Important sources of bacterial fecal pollution of environmental waters are also briefly indicated. In the last topic it is discussed which indicators of fecal pollution should be used in current drinking water microbiological analysis. It was concluded that safe drinking water for all is one of the major challenges of the 21st century and that microbiological control of drinking water should be the norm everywhere. Routine basic microbiological analysis of drinking water should be carried out by assaying the presence of *Escherichia coli* by culture methods. Whenever financial resources are available, fecal coliform determinations should be complemented with the quantification of enterococci. More studies are needed in order to check if ammonia is reliable for a preliminary screening for emergency fecal pollution outbreaks. Financial resources should be devoted to a better understanding of the ecology and behavior of human and animal fecal bacteria in environmental waters.

## 1. Drinking Water as a Vehicle of Diseases

Water is essential to life. An adequate, safe and accessible supply must be available to all. Improving access to safe drinking-water can result in significant benefits to health. Every effort should be made to achieve a drinking water quality as safe as possible [1].

Many people struggle to obtain access to safe water. A clean and treated water supply to each house may be the norm in Europe and North America, but in developing countries, access to both clean water and sanitation are not the rule, and waterborne infections are common. Two and a half billion people have no access to improved sanitation, and more than 1.5 million children die each year from diarrheal diseases [2]. According to the WHO, the mortality of water associated diseases exceeds 5 million people per year. From these, more that 50% are microbial intestinal infections, with cholera standing out in the first place.

In general terms, the greatest microbial risks are associated with ingestion of water that is contaminated with human or animal feces. Wastewater discharges in fresh waters and costal seawaters are the major source of fecal microorganisms, including pathogens [1–4].

Acute microbial diarrheal diseases are a major public health problem in developing countries. People affected by diarrheal diseases are those with the lowest financial resources and poorest hygienic facilities. Children under five, primarily in Asian and African countries, are the most affected by microbial diseases transmitted through water [5].

Microbial waterborne diseases also affect developed countries. In the USA, it has been estimated that each year 560,000 people suffer from severe waterborne diseases, and 7.1 million suffer from a mild to moderate infections, resulting in estimated 12,000 deaths a year [6]. The most important bacterial diseases transmitted through water are listed in Table 1.

## 2. Cholera

### 2.1. The Genus Vibrio

*Vibrio* are small, curved-shaped Gram-negative rods, with a single polar flagellum. Vibrios are facultative anaerobes capable of both fermentative and respiratory metabolism. Sodium stimulates growth of all species and is an absolute requirement for most. Most species are oxidase-positive and reduce nitrate to nitrite. Cells of certain species (*V. cholerae*, *V. parahaemolyticus* and *V. vulnificus*) have pili (fimbriae), structures composed of protein TcpA. TcpA formation is co-regulated with cholera toxin expression and is a key determinant of *in vivo* colonization (see below) [7,8].

Several *Vibrio* species can infect humans (Table 2). *V. cholerae* is, by far, the most important of these species. *V. alginolyticus* has been isolated from several types of soft tissue infections.

*V. fluvialis*, *Grimontia hollisae* (*V. hollisae*), and *V. mimicus* can cause diarrhea or infections of the gastrointestinal tract. *V. furnissii* has been isolated from a few individuals with diarrhea, but there is no evidence that it can actually cause this pathology. *V. parahaemolyticus* is a well-documented causal agent of acute food-borne gastroenteritis, particularly in Japan and South East Asia. Cases are associated with the consumption of raw or undercooked shellfish such as oysters, shrimp, crabs, and lobster. *V. vulnificus* is an important cause of (often fatal) septicemia and wound infections. Other vibrios, namely *Allivibrio fischeri* (*Vibrio fischeri*) and *V. natriegens*, have no relation with humans [7,8].

Vibrios are primarily aquatic bacteria. Species distribution depends on sodium concentration and water temperature. Vibrios are very common in marine and estuarine environments, living free or on the surfaces and in the intestinal contents of marine animals. Species with a low sodium requirement are also found in freshwater habitats [7,8].

### 2.2. The Species Vibrio Cholerae

*Vibrio cholerae* cells can grow at 40 °C with pH 9–10. The growth is stimulated by the presence of sodium chloride. *Vibrio cholerae* is a very diverse bacterial species (Table 3). It is divided in ca. 200 serovarieties, characterized by the structure of the lipopolysaccharide (LPS) (O antigens). Only serovarieties O1 and O139 are involved in “true” cholera. Some other serovarieties can cause gastroenteritis, but not cholera. The distinction between Classical and El Tor biotypes is based on biochemical and virological characteristics [1,7,8,10,11].

### 2.3. Cholera

#### 2.3.1. Characterization of the disease

The incubation period for cholera is ca. 1–3 days. The disease is characterized by an acute and very intense diarrhea that can exceed one liter per hour. Cholera patients feel thirsty, have muscular pains and general weakness, and show signs of oliguria, hypovolemia, hemoconcentration, followed by anuria. Potassium in blood drops to very low levels. Patients feel lethargic. Finally, circulatory collapse and dehydration with cyanosis occurs [7].

The severity of the disease depends on several factors: (1) personal immunity: this may be conferred by both previous infections and by vaccines; (2) inoculum: the disease only occurs after ingestion of a minimum amount of cells, ca. 10^8^ [1,7,8,10,11]; (3) The gastric barrier: *V. cholera* cells likes basic media and therefore the stomach, normally very acidic, is an adverse medium for bacterial survival. Patients consuming anti-acidic medications are more susceptible to infection than healthy people; (4) blood group: for still unknown reasons, people with O-group blood are more susceptible than others [1,7,8,10,11].

In the absence of treatment, the mortality of cholera-patients is ca. 50%. It is mandatory to replace not only lost water but also lost salts, mainly potassium. In light dehydrations, water and salts can be orally-administered, but in severe conditions, rapid and intravenous-administration is obligatory. The most efficient antibiotic is currently doxicyclin. If no antibiotic is available for treatment, the administration of water with salts and sugar can, in many cases, save the patient and help in the recovery [1,7,8,10,11].

There are two main determinants of infection: (1) the adhesion of the bacterial cells to the intestinal mucous membrane. This depends on the presence of pili and adesins at the cell’s surface; (2) the production of cholera toxin [1,7,8,10,11].

#### 2.3.2. Cholera toxin

Cholera toxin is an exotoxin with a very precise action on target cells. The toxin attaches to a specific receptor (ganglioside Gl) on the cell membrane of intestinal cells and activates the enzyme adenylate cyclase. This results in a non-stop degradation of internal ATP, with release of cAMP and inorganic phosphate. The rise in the internal concentration of cAMP causes an efflux of water, sodium, potassium, chloride and carbonate ions from the cells of the mucous membrane, and this is the main cause of diarrhea [7].

#### 2.3.3. Cholera pandemics and the emergence of El Tor biotype and O139 serovariety. New facts about cholera epidemiology

Cholera has been a well known disease since the 19th century. In the 19th and 20th centuries, seven major pandemics are recognized. The first six pandemics occurred during the following periods: 1st: 1816–1826, 2nd: 1829–1851, 3rd: 1852–1860, 4th: 1863–1875, 5th: 1881–1896, 6th: 1899–1923. These pandemics all started in Asia, passed through Europe and then reached South America. The Classical biotype was involved. The seventh pandemic, still in course, started in 1961 in the Celebes Isles, in Asia. In the 1960s, the disease spread through Asia, in the 1970s reached the Middle East and Africa, and in 1991 streaked violently across South America. Now El Tor has replaced the Classical biotype. El Tor biotype had been detected before, in 1905, but only in the development of the seventh pandemic did this biotype replace the Classical one and become dominant [1,7,8,10,11].

In 1992, a new serovariety (O139), which was coined the Bengal serovariety, was detected for the first time in Bangladesh. This new serovariety quickly spread to India and to southeastern Asia, displacing O1. Although serovariety O1 El Tor has reappeared in 1994 and 1995, the Bengal serovariety still remains the dominant one. The illness caused by serovarieties O139 and O1 are indistinguishable [8,12,13].

In 1991, the seventh pandemic entered South America through the coastal area of Peru. On 23 January, in Chancay, north Peru, *Vibrio cholerae* O1 El Tor was isolated from patients with cholera symptoms, confirming the disease. In this region, between 24 January and 9 February, 1,859 people were hospitalized and 66 died. From Peru, the disease spread rapidly to other countries in South America. Two routes have been proposed for the entrance of the bacterium in Peru: (1) ballast water from a boat coming from Asia; (2) the El Niño current may have transported zooplankton harboring *V. cholerae* cells. Shellfish and fish nourishing on this zooplankton became contaminated and the bacterium was transmitted to humans who ate these marine foods [14–17].

The misfortune of people who died in the first months of this disastrous South American cholera epidemic appeared to have unleashed scientists to study the disease harder and, indeed, important epidemiological studies were carried out during this outbreak. These studies confirmed that contaminated uncooked food and beverages can also be a vehicle for transmission of cholera [18].

#### 2.3.4. Genes for toxin and pili protein production

The genes responsible for toxin production are harbored in the CTXΦ segment (7–9.7 kb) of the chromosome (only in toxigenic strains). The CTXΦ segment carries at least six genes. In addition to the gene encoding cholera toxin production, this segment (virulence cassette) include an accessory cholera toxin (*ace*), a zonula occludens toxin (*zot*), core encoded pilin (*cep*), and an open reading frame of unknown function. During the replication of the chromosome, the CTXΦ fragment can form an autonomous copy and this can constitute an independent plasmid. The plasmid can give rise to virus-like particles—CTXΦ bacteriophages, which can infect non-toxigenic strains. The CTXΦ segment incorporates into the chromosome of the infected cells which became toxigenic. This process was demonstrated *in vitro* in cell suspensions and *in vivo* in the gut of the rat [8,13,19,20].

Epidemic and pandemic strains of *V. cholerae* contain another chromosomal segment designated as VPI. VPI is 39.5 kb in size and contains two ToxR-regulated genes: a regulator of virulence genes (ToxT) and a gene cluster containing colonization factors, including the toxin co-regulated pili (TCP). The tcp gene encodes for the 20.5-kDa TcpA pili protein. This VPI segment appears to be transferable from *V. cholerae* O1 to non-O1 strains. *V. cholerae* O139 strains, like O1, carry the structural genes encoded by the CTX operon and TCP. *V. cholerae* strains non-O1 or O139 normally lack cholera toxin genes and have never been found to carry TCP [8].

#### 2.3.5. Ecology of the bacterium and the cycle of the disease

*V. cholerae* non-O1 or O139 strains are common in the environment, especially in estuaries. They have been isolated from many estuarine animals such as birds, frogs, fishes and shellfish, and survive and multiply on the surface of phytoplankton and zooplankton cells [8,21].

*V. cholerae* O1 and O139 strains are isolable from the environment only in epidemic areas. They survive in the cultivable state in water and aquatic and marine organisms for a considerable period of time [8,12,22–24]. When *V. cholerae* cells face adverse environmental conditions, they reduce cell size, became coccoid and enter a dormant stage inside exopolysaccharide biofilms. Cells display a certain metabolism, but are not able to growth and multiply on the surface of agarized media and give rise to colonies. Cells in this viable but non-culturable state retain viability as well as the potential for pathogenicity for significant periods of time [25–27].

Viable but non-culturable cells can leave their dormant stage and multiply again, resulting in an explosion of their concentration in the environment. Since the presence of non-toxigenic strains is common in aquatic milieu, especially in estuaries, if a horizontal transfer of cholera exotoxin producing genes occurs between toxigenic and non-toxigenic strains, the number of toxigenic cells in the environment can rise rapidly and pronouncedly. The episodic nature and the sudden appearance of violent cholera outbreaks, followed by a rapid slowing down, are probably related with these phenomena.

## 3. Salmonellosis

### 3.1. The Genus Salmonella. Pathogenicity of Main Serovars

The genus *Salmonella* was designated by Lignières in 1900 [28,29]. Antigenic analysis began when Castellani described, in 1902, a method for absorbing antisera. The first antigenic scheme for *Salmonella* was published by White in 1926, and subsequently developed extensively by Kauffmann, in two classical works published in 1966 and 1978 [28,29]. The Kauffmann-White antigenic scheme contained, by 1988, about 2,250 different serovars [28,29].

The genus *Salmonella*, a member of the family *Enterobacteriaceae*, include Gram-negative motile straight rods. Cells are oxidase-negative and catalase-positive, produce gas from D-glucose and utilize citrate as a sole carbon source. Salmonellae have several endotoxins: antigens O, H and Vi [28,29].

The concept “one serovar-one species”, in use for many years, is no longer acceptable. The taxonomy and nomenclature of the genus *Salmonella* has been subject of debate since Le Minor and Popoff proposed changes in a paper published in 1987. The issue was settled by a decision of the International Committee on the Systematics of Prokaryotes and published in 2005. The current taxonomy of the genus is presented in Table 4. According to the rules of bacterial nomenclature, the names of the serovars are not italicized and the first letter must be a capital [28–30].

*S. enterica* subsp. *enterica* serovar Enteritidis is the most frequently isolated serovar from humans all over the world. However, locally, other serovars can be predominant. In the period 1994–2004, Tunisia was exposed to salmonellosis outbreaks in 1997, 1999, 2002 and 2004. In 1997, salmonellosis outbreak was caused by serovar Mbandaka. In 1999, three salmonellosis outbreaks were reported from hospitals located in three different regions. Each outbreak was associated with a different serotype: Mbandaka, Livingstone and Typhi Vi+. In 2002, a *S. enterica* subsp. *enterica* serovar Livingstone infection occurred in the same hospital that reported an outbreak caused by serovar Typhi Vi+ in 1999, but in a different unit. In that year, the Livingstone serovar jumped to the first position in human infection in Tunisia. In 2004, a second outbreak by serovar Typhi Vi+ was reported. The source of isolation was a fermented juice traditionally extracted from palm-tree [31].

### 3.2. Characterization of the Diseases

Salmonellae pathogenic to humans can cause two types of salmonellosis: (1) typhoid and paratyphoid fever (do not confuse with typhus, a disease caused by a rickettsia); (2) gastroenteritis [28]. Low infective doses (less than 1,000 cells) are sufficient to cause clinical symptoms. Salmonellosis of newborns and infants presents diverse clinical symptoms, from a grave typhoid-like illness with septicemia to a mild or asymptomatic infection. In pediatric wards, the infection is usually transmitted by the hands of staff [29].

Food-borne *Salmonella* gastroenteritis are frequently caused by ubiquitous *Salmonella* serovars such as Typhimurium. About 12 h following ingestion of contaminated food, symptoms (diarrhea, vomiting and fever) appear and last 2–5 days. Spontaneous cure usually occurs. *Salmonella* may be associated with all kinds of food. Prevention of *Salmonella* food-borne infection relies on avoiding contamination (improvement of hygiene), preventing multiplication of *Salmonella* in food (constant storage of food at 4 °C), and use of pasteurization (milk) or sterilization when possible (other foods). Vegetables and fruits may carry *Salmonella* when contaminated with fertilizers of fecal origin, or when washed with polluted water [28].

The incidence of typhoid fever decreases when the level of development of a country increases (*i.e.*, controlled water sewage systems, pasteurization of milk and dairy products). Where these hygienic conditions are missing, the probability of fecal contamination of water and food remains high and so is the incidence of typhoid fever [29].

### 3.3. Ecology of Salmonellae and the Cycle of Salmonellosis

The principal habitat of *Salmonella* is the intestinal tract of humans and animals [28]. Salmonellae are constantly found in environmental samples, because they are excreted by humans, pets, farm animals, and wild life. Municipal sewage, agriculture pollution, and storm water runoff are the main sources of these pathogens in natural waters [1,32]. Salmonellae do not seem to multiply significantly in the natural environment, but they can survive several weeks in water and in soil if conditions of temperature, humidity, and pH are favorable [28].

Salmonellae isolated from environmental sources are predominantly non-Typhi or Paratyphi serovars. In a study carried out in Tunisia during 1994–2004, *S. enterica* subsp. *enterica* serovars Anatum, Enteritidis and Corvallis were the most common serotypes isolated from food. The great majority of the strains were isolated from poultry, red meat, milk and dairy products, vegetables and fruits. From environmental sources, 73% of the isolates were from tap water. Serovars Corvallis, Enteritidis, and Anatum were the commonest [31]. Arvanitidou *et al.* [32] reported a comparative study carried out in Rivers Aliakmon and Axios, in northern Greece, during a 1-year period, from May 2002 to April 2003. A total of 29 *Salmonella* species were recovered from the water samples. Many of the isolated *Salmonella* serovars were of non-human animal origin such as Mbantaka, Virchow, Hadar, Infantis and Senftenberg, commonly isolated from poultry farm.

Unlike cholera, humans infected with salmonellae can carry the bacteria in the gut without signs of disease. Infected humans can harbor the bacteria for considerable periods of time. About 5% of patients clinically cured from typhoid fever remain carriers for months or even years. These people can be chronic holders of the bacterium in the gut, and constitute the main reservoir of the bacteria in the environment [29].

The salmonellosis cycle in the environment can involve shellfish. Salmonellae survive sewage treatments if suitable germicides are not used in sewage processing. If effluent from the sewage plant passes into a coastal area, edible shellfish (mussels, oysters) can become contaminated. Shellfish concentrate bacteria as they filter several liters of water per hour. Ingestion by humans of these seafoods (uncooked or superficially cooked) may cause typhoid fever or other salmonellosis. Evidence of such a cycle has been obtained by the use of strain markers, including phage typing [29].

## 4. Shigellosis or Bacillary Dysentery

### 4.1. The Genus Shigella

*Shigella* are Gram-negative, non-sporeforming, non-motile, straight rod-like members of the family *Enterobacteriaceae*. Cells ferment sugars without gas production. Salicin, adonitol and myo-inositol are not fermented. Cells do not utilize citrate, malonate and acetate as sole carbon source and do not produce H_2_S. Lysine is not decarboxylated. Cells are oxidase-negative and catalase-positive. Members of the genus have a complex antigenic pattern, and taxonomy is based on their somatic O antigens [1,33,34].

### 4.2. Characterization of the Disease

The incubation period is 1–4 days. The disease usually begins with fever, anorexia, fatigue and malaise. Patients display frequent bloody stools of small volume (sometimes grossly purulent) and abdominal cramps. Twelve to 36 hours later, diarrhea progresses to dysentery, blood, mucus and pus appearing in feces that decreases in volume (no more than 30 mL of fluid per kg per day) [34–36].

Although the molecular basis of shigellosis is complex, the initial step in pathogenesis is penetration of the colonic mucosa. The resulting focus of *Shigella* infection is characterized by degeneration of the epithelium and by an acute inflammatory colitis in the lamina propria. Ultimately, desquamation and ulceration of the mucosa cause leakage of blood, inflammatory elements, and mucus into the intestinal lumen. Under these conditions the absorption of water by the colon is inhibited and the volume of stool is dependent upon the ileocecal flow. As a result, the patient will pass frequent, scanty, dysenteric stools [37,38].

In order for *Shigella* to enter an epithelial cell, the bacterium must first adhere to its target cell. Generally, the bacterium is internalized via an endosome, which it subsequently lyses to gain access to the cytoplasm where multiplication occurs [37,38].

### 4.3. Virulence Factors

*S. dysenteriae* serotype 1 produces high levels of a cytotoxic Shiga toxin. *S. sonnei* and *S. flexneri* produce much lower amounts of this toxin. Shiga toxin binds to Galotl-4Galp (galabiose) glycolipid receptors and inhibits mammalian protein synthesis by cleaving the N-glycosidic bond at adenine 4324 in 28S rRNA. The toxic mechanism is identical to that of the plant toxin ricin, produced by *Ricinus communis*. Shigella also release a LPS endotoxin (O antigens), that cause an inflammatory response [37,38].

*Shigella* 180- to 230-kb plasmids encode genes essential for virulence, namely for: production of adhesins involved in the adherence of bacteria onto the surface of target epithelial cells; production of invasion plasmid antigens (Ipa) that have a direct role in the *Shigella* invasion process; transport or processing functions that ensure the correct surface expression of the Ipa proteins; induction of endocytic uptake of bacteria and disruption of endocytic vacuoles; regulation of plasmid-encoded virulence genes [37,38].

*Shigella* emerged from *E. coli* during evolution. The acquisition and evolution of the pathogenicity island which encodes all of the genes required for cell invasion and phagolysosomal lysis, permitted a major alteration in pathogenesis [37,38].

### 4.4. Risk Factors

Many studies have identified risk factors and protective effects for shigellosis incidence and fatality. Despite gradual improvements in water supply, shigellosis continues to be endemic among the disadvantaged populations living in the tropics, often among displaced populations following natural disasters and political crises. In Guatemala, young children, the elderly, and 15–44-year-old males were found to be most susceptible to *S. dysenteriae* serotype 1. In Sierra Leone, the attack rate was higher among children younger than 5 years of age than in the rest of the population. In rural Bangladesh, shigellosis was most common in children aged 1–2 years and in people 60 years or older. In Dhaka, Bangladesh, it was found that shigellosis mortality was most common in severely malnourished people of all ages, in children under 2 who were not being breastfed, and in all children under 1. In a 3-year study carried out in Matlab, Bangladesh, during 1992 to 1994, it was found that the incidence of *S. dysenteriae* serotype 1 and *S. flexneri* was highest in children under 2 followed by children from 2 to 5. The location of *S. dysenteriae* serotype 1 risk varies in time but *S. flexneri* risk areas were persistent in time. Neighborhoods near bazaars with many non-septic latrines were at highest risk for *S. dysenteriae* serotype 1. *S. flexneri* was most common in flood-controlled areas. It was concluded that *S. dysenteriae* serotype 1 risk was more related to hygiene and sanitation whereas *S. flexneri* was more related to the environment [35].

### 4.5. Shigellosis through the World

The total number of *Shigella* episodes that occur each year throughout the World is estimated to be 164.7 million, including 163.2 million cases in developing countries, 1.1 million of which result in death. Children under 5 account for 61% of all deaths attributable to shigellosis [35,36].

*Shigella* species are not uniformly distributed in the world. *S. dysenteriae* is usually found in densely populated areas of South America, Africa and Asia. Infections usually result in significant epidemic outbreaks. Serotype 1 has been distinguished by both its virulence and its ability to produce ravaging epidemics. It predominates in India, Malaysia and Guatemala. Serotype 2 predominates in Yemen and Nigeria. *S. flexneri* is usually found in areas where endemic shigellosis occurs. *S. boydii* occurs sporadically, except in the Indian subcontinent where it was first identified. *S. sonnei* usually occurs in Western developed countries, such as France and USA [35,36].

Important epidemics were reported in the last decades: (1) in 1970 in Central America where 112,000 people were affected and 13,000 died; (2) in 1985, in Texas (USA), 5,000 people became infected after ingestion of contaminated lettuce; (3) in May–June 1994, domestic cases of *S. sonnei* infection were detected in several European countries, including Norway, Sweden, and the United Kingdom. Epidemiological evidence incriminated imported iceberg lettuce as the vehicle of transmission; (4) in 1996, in Paris, with 153 reported patients [33].

### 4.6. Ecology of Shigellae and the Cycle of Shigellosis

*Shigella* is typically an inhabitant of the intestinal tract of humans and other primates [1,33,34,36,39]. It is typically spread by fecal-contaminated drinking water or food, or by direct contact with an infected person. In water, shigellae can survive for at least six months at room temperature, and this high survival favors transmission through water. Flies have been implicated on the transmission of *Shigella* cells from human feces to foods. The hand is an important vehicle for transmission of shigellosis, since *S. dysenteriae* serotype 1 cells survives for up to one hour on a human’s skin and a very small inoculum is required to unchain infection and disease. Indeed, studies on American volunteers experimentally infected with *Shigella* have shown that as few as one hundred *Shigella* cells given orally cause the disease in 25–50% of the cases. Resistance of *Shigella* to gastric juice certainly accounts, although not exclusively, for this high infectivity [36,40]. Asymptomatic and inappropriately-treated patients with shigellosis can harbor the bacteria in the gut and these appear to be the main reservoirs of the bacteria in the environment [41].

Recent reports on the ecology of shigellae have brought new elements for the understanding of the cycle of the disease. In environmental waters of regions with high numbers of shigellosis’ cases, it has been found that, although numbers of cultivable cells were low, genetic elements such as plasmids and genetic fragments with bacteriophage origin, could be detected. Many of the genes that code for exotoxin production are precisely found in these genetic elements. These results suggest that the sudden rise of the number of virulent strains in the environment can result from the incorporation, by cells with reduced virulence, of this type of genetic elements present in the waters. If this hypothesis is confirmed, there is a certain similarity between the cholera and the shigellosis cycles in the environment. It remains to be elucidated if shigellae can also exist in environmental waters in a viable but non-culturable state, as vibrios [41].

## 5. Pathogenic *Escherichia coli* Strains

*E. coli* strains isolated from intestinal diseases have been grouped into at least six different main groups, based on epidemiological evidence, phenotypic traits, clinical features of the disease and specific virulence factors. From these, enterotoxigenic (ETEC, namely O148), enterohemorrhagic (EHEC, namely O157) and enteroinvasive serotypes (EIEC, namely O124) are of outstanding importance and can be transmitted through contaminated water [42,43].

### 5.1. Enterotoxigenic E. coli (ETEC) Strains

Enterotoxigenic *E. coli* (ETEC) serotypes can cause infantile gastroenteritis. The number of reports of their occurrence in developed countries is comparatively small, but it is an extremely important cause of diarrhea in the developing world, where there is no adequate clean water and poor sanitation. In developing countries, these strains are the most commonly isolated bacterial enteropathogen in children below 5 years of age, and account for several hundred million cases of diarrhea and several ten of thousand deaths each year [42–44].

Disease caused by ETEC follows ingestion of contaminated food or water and is characterized by profuse watery diarrhea lasting for several days that often leads to dehydration and malnutrition in young children [42–44]. ETEC also are the most common cause of “travelers’ diarrhea” that affects individuals from industrialized countries travelling to developing regions of the World [42–44].

### 5.2. Enterohemorrhagic E. coli (EHEC) Strains

Reported outbreaks had been associated mainly with the consumption of contaminated foods, such as raw or undercooked ground meat products and raw milk. The primary reservoir of this bacterium has been found to be healthy cattle [42,45,46].

*E. coli* serotype O157:H7 causes abdominal pain, bloody diarrhea, and hemolytic uremic syndrome. This bacterium produces Shiga-like toxins. The incubation period is 3–4 days, and the symptoms occur for 7–10 days. It is estimated that 2–7% of *E. coli* O157:H7 infections result in acute renal failure [42,45,46].

Although *E. coli* O157:H7 is not usually a concern in treated drinking water, outbreaks involving consumption of drinking water contaminated with human sewage or cattle feces have been documented. An increasing number of outbreaks are associated with the consumption of fruits and vegetables (sprouts, lettuce, coleslaw, salad) contaminated with feces from domestic or wild animals at some stage during cultivation or handling. EHEC has also been isolated from bodies of water (ponds, streams), wells and water troughs, and has been found to survive for months in manure and water-trough sediments [45,46].

Person-to-person contact is an important mode of transmission through the oral-fecal route. An asymptomatic carrier state has been reported, where individuals show no clinical signs of disease but are capable of infecting others [45,46].

### 5.3. Enteroinvasive E. coli (EIEC) Strains

Enteroinvasive *E. coli* (EIEC) behave in many respects like shigellae. They are capable of invading and multiplying in the intestinal epithelial cells of the distal large bowel in humans. The illness is characterized by abdominal cramps, diarrhea, vomiting, fever, chills, a generalized malaise, and the appearance of blood and mucus in the stools of infected individuals. [42,43,47].

EIEC strains were isolated, for instance, from 28 subjects in the Jesreel district of Israel during a peak period for dysentery. An investigation in Croatia showed that *E. coli* O124 could frequently be isolated from cases of gastroenteritis, enterocolitis, and dysentery. The dysentery was more common among the older age groups, while the two other types of disease occurred equally in all age groups. A 1985 survey was carried out in Bankok, Thailand in which 410 children with diarrhea and an equal number of control children without diarrhea were examined for the presence of strains of *Shigella*, EIEC, and other pathogens. It was found that 17 of the children with diarrhea and six without yielded EIEC [42,43].

Any food contaminated with human feces from an ill individual, either directly or via contaminated water, could cause disease in others. Outbreaks have been associated with hamburger meat and unpasteurized milk [47].

## 6. Emerging Waterborne Bacterial Pathogens

The emerging pathogenic bacteria of concern outlined here have the potential to be spread through drinking water, but they do not correlate with the presence of *E. coli* or with other commonly used drinking water quality indicators, such as coliform bacteria. In most cases, there are no satisfactory microbiological indicators of their presence. More studies are needed in order to understand the real significance and dimension of the diseases caused by water contaminated with these bacteria, and the ecology of these pathogens [45].

### 6.1. Mycobacterium Avium Complex (Mac)

The *Mycobacterium avium* complex (Mac) consists of 28 serovars of two distinct species: *Mycobacterium avium* and *Mycobacterium intracellulare.* The importance of Mac organisms was recognized with the discovery of disseminated infection in immunocompromised people, particularly people with HIV and AIDS. Members of MAC are considered opportunistic human pathogens [45,48].

Mac organisms have been identified in a broad range of environmental sources, including marine waters, rivers, lakes, streams, ponds, springs, soil, piped water supplies, plants, and house dust. Mac organisms have been isolated from natural water and drinking water distribution systems in the USA [45,49,50].

The ubiquitous nature of Mac organisms results from their ability to survive and grow under varied conditions. Mac organisms can proliferate in water at temperatures up to 51 °C and can grow in natural waters over a wide pH range [45]. These mycobacteria are highly resistant to chlorine and the other chemical disinfectants used for the treatment of drinking-water. Standard drinking-water treatments will not eliminate Mac organisms but, if operating satisfactorily, will significantly reduce the numbers that may be present in the source water to a level that represents a negligible risk to the general population. The entryway of these mycobacteria in distribution systems is through leaks. Growth of Mac organisms in biofilms is probably important for their continuous presence in distribution systems. Slow growing mycobacteria can be found at densities greater than 4,000 per cm^2^ in the surface biofilm, creating a potentially high level of exposure [48].

The symptoms encountered with Mac infections result from colonization of either the respiratory or the gastrointestinal tract, with possible dissemination to other locations in the body. Exposure to Mac organisms may occur through the consumption of contaminated food, the inhalation of air with contaminated soil particles, or contact with or ingestion, aspiration, or aerosolization of potable water containing the organisms [45].

With respect to water supplies, infection with *M. avium* and *M. intracellulare* has been well documented. Unlike gastrointestinal pathogens, where *E. coli* can be used to indicate their potential presence, no suitable indicators have been identified to signal increasing concentrations of Mac organisms in water systems [45].

### 6.2. Helicobacter Pylori

*Helicobacter pylori* has been cited as a major etiologic agent for gastritis and has been implicated in the pathogenesis of peptic and duodenal ulcer disease and gastric carcinoma. However, most individuals that are infected by this pathogen remain asymptomatic [45].

Using culture-based methods, *H. pylori* has not been isolated from environmental sources, including water [45,51]. On the contrary, molecular methods have been successful in detecting this pathogen. Fluorescence *in situ* hybridization has been successfully used to detect this pathogen in drinking water distribution systems and other water bodies. Polymerase chain reaction has also been used to detect the presence of *H. pylori* DNA in drinking water, especially associated with biofilms [45,51,52]. In drinking-water biofilms, *H. pylori* cells rapidly lose culturability, entering a viable but non-culturable state. In these biofilms, cells can persist for more than one month, with densities exceeding 10^6^ cells per cm^2^ [51].

How the organism is transmitted is still not fully understood. However, the fact that it has been recovered from saliva, dental plaques, the stomach, and fecal samples strongly indicates oral-oral or fecal-oral transmission. Water and food appear to be of lesser direct importance, but they can still play a significant role in situations with improper sanitation and hygiene [45].

### 6.3. Aeromonas Hydrophyla

In recent years, *A. hydrophila* has gained public health recognition as an opportunistic pathogen. It has been implicated as a potential agent of gastroenteritis, septicemia, meningitis, and wound infections. It can play a significant role in intestinal disorders in children under five years old, the elderly, and immunosuppressed people. [45,53,54].

*Aeromonas hydrophila* are Gram-negative, non-sporeforming, rod-shaped, facultative anaerobic bacilli belonging to the family *Aeromonadaceae*. Although *A. hydrophila* is usually the dominant species, other aeromonads, such as *A. caviae* and *A. sobria*, have also been isolated from human feces and from water sources [45,54].

*Aeromonas* species, including *A. hydrophila*, are ubiquitous in the environment. It is frequently isolated from food, drinking water, and aquatic environments [45,53,54]. In clean rivers and lakes, concentrations of *Aeromonas* spp. are usually around 10^2^ colony-forming units (CFU)/mL. Groundwaters generally contain less than 1 CFU/mL. Drinking water immediately leaving the treatment plant has been found to contain between 0 and 10^2^ CFU/mL. Drinking water in distribution systems can display higher *Aeromonas* concentrations, due to the growth in biofilms [45,55]. *Aeromonas* spp. have been found to grow between 5 °C and 45 °C [44,54]. *A. hydrophila* is resistant to standard chlorine treatments, probably surviving inside biofilms [56].

The common routes of infection suggested for *Aeromonas* are the ingestion of contaminated water or food or contact of the organism with a break in the skin. Drinking or natural mineral water can be a possible source of contamination for humans. No person-to-person transmission has been reported [45,54].

## 7. Microbiological Water Analysis

### 7.1. The Rationale of the Use of Fecal Indicator Bacteria

The most important bacterial gastrointestinal diseases transmitted through water are cholera, salmonellosis and shigellosis. These diseases are mainly transmitted through water (and food) contaminated with feces of patients. Drinking water can be contaminated with these pathogenic bacteria, and this is an issue of great concern. However, the presence of pathogenic bacteria in water is sporadic and erratic, levels are low, and the isolation and culture of these bacteria is not straightforward. For these reasons, routine water microbiological analysis does not include the detection of pathogenic bacteria. However, safe water demands that water is free from pathogenic bacteria [57].

The conciliation of the two needs was met by the discovery and testing of indicator bacteria. Water contaminated with pathogenic species also has the normal inhabitants of the human intestine. A good bacterial indicator of fecal pollution should fulfill the following criteria: (1) exist in high numbers in the human intestine and feces; (2) not be pathogenic to humans; (3) easily, reliably and cheaply detectable in environmental waters. Additionally, the following requisites should be met if possible: (4) does not multiply outside the enteric environment; (5) in environmental waters, the indicator should exist in greater numbers than eventual pathogenic bacteria; (6) the indicators should have a similar die-off behavior as the pathogens; (7) if human fecal pollution is to be separated from animal pollution, the indicator should not be very common in the intestine of farm and domestic animals [1,4,6,57,58]. The usefulness of indicator bacteria in predicting the presence of pathogens was well illustrated in many studies, namely by Wilkes *et al.* [59].

### 7.2. The Composition of Human and Animal Feces

Microbiological analysis of the human feces was important in order to structure and validate the use of fecal indicator bacteria in environmental waters. Bacteria present in feces are naturally derived from the microbiota of the human gastrointestinal tract.

Although bacteria are distributed throughout the human gastrointestinal tract, the major concentration of microbes and metabolic activity can be found in the large intestine. The upper bowel (stomach, duodenum, and jejunum) has a sparse microbiota with up to 10^5^ CFU/ml of contents. From the ileum on, bacterial concentrations gradually increase reaching in the colon 10^10^ to 10^11^ CFU/g [60].

It has been estimated that at least 500–1,000 different microbial species exist in the human gastrointestinal microbiota, although on a quantitative basis 10–20 genera usually predominate (Table 6). The total number of microbial genes in the human gastrointestinal tract has been estimated as 2–4 million. This represents an enormous metabolic potential which is far greater than that possessed by the human host [60,64].

The composition of feces from an individual is stable at genus level, but the species composition can vary markedly from day to day. The relative proportion of intestinal bacterial groups can vary between individuals [60,64].

The microflora of the human gastrointestinal tract is dominated by obligate anaerobes, which are ca. 10^3^ more abundant than facultative anaerobes. The main anaerobic genera are *Bacteroides*, *Eubacterium* and Bifidobacteria. These organisms account for ca. 90% of the cultivable human fecal bacteria. *Bacteroides* (mainly *B. thetaiotaomicron* and *B. vulgatus*) are the most abundant organism in the human feces and account for 20–30% of cultivable bacteria. The most abundant facultative anaerobes are Enterococci and *Enterobacteriaceae*. The main *Enterobacteriaceae* genera are *Escherichia*, *Citrobacter*, *Klebsiella*, *Proteus* and *Enterobacter. Citrobacter* and *Klebsiella* are present in most individuals although in low numbers. *Proteus* and *Enterobacter* are only present in a minority of humans [64].

A variety of molecular techniques have been used to study the microbial composition of the human gastrointestinal tract. Results yielded by these studies have shown that many microbes detected by molecular techniques are not isolable by conventional culture-based methods. The presence of high proportions of bifidobacteria detected by culture-based methods is not supported by the results of molecular-based studies. However, the results of molecular-based approaches support many of the findings derived from culture-based methods: the dominance of the obligate anaerobes over facultative anaerobes; the presence of high counts of *Bacteroides*, *Clostridium* and *Eubacterium* [64].

Anaerobic bacteria such as *Bacteroides* and *Eubacterium* are not easily cultured by conventional techniques since require incubation chambers with nitrogen atmosphere. *Bifidobacterium* and *Lactobacillus* tolerate some oxygen but are fastidious bacteria growing very slowly in culture media. Therefore, these four genera are not adequate to be used as indicators of fecal pollution (the introduction of molecular techniques may improve the situation). *Citrobacter*, *Klebsiella* and *Enterobacter* are present in low numbers in the human intestine and are widespread in environmental waters, and therefore are also not suitable as indicators of fecal pollution. *Clostridium*, *Streptococcus* and *Escherichia* do not suffer from these drawbacks. Therefore, their suitability as fecal indicators has been tested since several decades.

### 7.3. Fecal Bacteria in Their Hosts and in the Environment

#### 7.3.1. Bacteroides

The traditional genus *Bacteroides* included Gram-negative, non-sporeforming, anaerobic pleiomorphic rods. Many species have been transferred to other genera—*Mitsuokella, Porphyromonas, Prevotella, Ruminobacter*. Bacteroides are the most abundant bacteria in human feces. In animal feces, on the contrary, *Bacteroides* are present at low numbers. Although anaerobic, *Bacteroides* are among the most tolerant to oxygen of all anaerobic human gastrointestinal species. *B. thetaiotaomicron* is one of the most abundant species in the lower regions of the human gastrointestinal tract. *Bacteroides* have a high pathogenic potential and account for approximately two-thirds of all anaerobes isolated from clinical specimens. The most frequently isolated species has been *B. fragilis*. The survival of *Bacteroides* in environmental waters is usually much lower than the survival of coliforms [64,65].

#### 7.3.2. Eubacterium

The traditional genus *Eubacterium* included anaerobic non-sporeforming Gram-positive rods. Some species have been transferred to other genera—*Actinobaculum*, *Atopobium*, *Collinsella*, *Dorea*, *Eggerthella*, *Mogibacterium*, *Pseudoramibacter* and *Slackia*. Cells are not very aerotolerant. Species isolated from the human gastrointestinal tract include: *E. barkeri*, E*. biforme*, *E. contortum*, *E. cylindrioides*, *E. hadrum*, *E. limosum*, *E. moniliforme, E. rectal* and *E. ventricosum* [64].

#### 7.3.3. Bifidobacterium

Bifidobacteria are Gram-positive, non-sporeforming, pleiomorphic rods. Bifidobacteria are anaerobic (some species tolerate oxygen in the presence of carbon dioxide) or facultative anaerobic. The optimum growth temperature is 35–39 °C. The genus *Bifidobacterium* contains ca. 25 species, most of which have been detected in the human gastrointestinal tract [64–66].

Bifidobacteria are present in high numbers in the feces of humans and some animals. Several *Bifidobacterium* species are specific either for humans or for animals. *B. cuniculi* and *B. magnum* have only been found in rabbit fecal samples, *B. gallinarum* and *B. pullorum* only in the intestine of chickens and *B. suis* only in piglet feces. In human feces, the species composition changes with the age of the individual. In the intestine of infants *B. breve* and *B. longum* generally predominate. In the adult, *B. adolescentis*, *B. catenulatum*, *B. pseudocatenulatum* and *B. longum* are the dominant species. In both human and animal feces, bifibobacteria are always much more abundant than coliforms [64–66].

Bifidobacteria have been found in sewage and polluted environmental waters, but appears to be absent from unpolluted or pristine environments such as springs and unpolluted soil. This results from the fact that upon introduction into the environment, bifidobacteria decrease appreciably in numbers, probably due to their stringent growth requirements. Bifidobacteria grow poorly below 30 °C and have rigorous nutrient requirements. Reports on the survival of bifidobacteria in environmental waters indicate that their survival is lower than that of coliforms [64–66].

The presence of bifidobacteria in the environment is therefore considered an indicator of fecal contamination. Since some species are specific for humans and animals, the identification of *Bifidobacterium* species present in the polluted water could, in principle, provide information on the origin of fecal pollution [64–66].

A study carried out in a highly contaminated stream near Bologna, Italy, revealed that *B. adolescentis*, *B. catenulatum*, *B. longum*, *B. pseudocatenulatum* and *B. thermophilum* were the most representative species, whereas *B. angulatum*, *B. animalis* subsp. *animalis (B. animalis)*, *B. breve*, *B. choerinum*, *B. minimum*, *B. pseudolongum* subsp. *globosum* (*B. globosum*) and *B. subtile* occurred only in low numbers [66].

Bifidobacteria are the less studied of all fecal bacteria, due to the technical difficulties in their isolation and cultivation. Other Gram-positive bacteria, such as *Streptococcus* and *Lactobacillus*, which may occur in higher numbers than bifidobacteria, can inhibit their growth. Although selective media has been designed for the isolation of bifidobacteria from environmental waters, the outcome is still unsatisfactory, with appreciable numbers of false positives and low recovery percentages [64–66].

#### 7.3.4. Clostridia

The genus *Clostridium* is one of the largest genera of the prokaryotes containing 168 validly published species. From these, 77 (including *C. perfringens*) are considered to belong to a united group—*Clostridium sensu stricto* [64,67,68].

Clostridia are Gram-positive rods, forming endospores. Most of the clostridial species are motile with peritrichous flagellation. Cells are catalase-negative and do not carry out a dissimilatory sulphate reduction. Clostridia usually produce mixtures of organic acids and alcohols from carbohydrates and proteins. Many species are saccharolytic and proteolytic. Some species fix atmospheric dinitrogen [64,67,68].

The genus *Clostridium* includes psychrophilic, mesophilic, and thermophilic species. The major role of these organisms in nature is in the degradation of organic material to acids, alcohols, CO_2_, H_2_, and minerals. Frequently, a butyric acid smell is associated with the proliferation of clostridia. The ability to form spores that resist dryness, heat, and aerobic conditions makes the clostridia ubiquitous [64,67,68].

Most species are obligate anaerobic, although tolerance to oxygen occurs. Oxygen sensitivity restricts the habitat of the clostridia to anaerobic areas or areas with low oxygen tensions. Growing and dividing clostridia will, therefore, not be found in air saturated surface layers of lakes and rivers or on the surface of organic material and soil. Clostridial spores, however, are present with high probability in these environments, and will germinate when oxygen is exhausted and when appropriate nutrients are present [64,67,68].

*C. perfringens* ferment lactose, sucrose and inositol with the production of gas, produce a stormy clot fermentation with milk, reduce nitrate, hydrolyze gelatin and produce lecithinase and acid phosphatase. The species is divided into five types, A to E, on the basis of production of major lethal toxins [68,69].

*C. perfringens* appears to be a universal component of the human and animal intestine, since has been isolated from the intestinal contents of every animal that has been studied. Humans carry *C. perfringens* as part of the normal endogenous flora. The main site of carriage is the distal gastrointestinal tract. The principal habitats of type A are the soil and the intestines of humans, animals, and birds. Types B, C, D, and E appears to be obligate parasites of animals and occasionally are found in humans [68,69].

*Clostridium perfringens* is the most frequently isolated *Clostridium* in clinical microbiology laboratories, although it seldom causes serious infections. *C. perfringens* is isolated from infections in humans and the organism most commonly found in gas gangrene in humans. *C. perfringens* is most commonly isolated from infections derived from the colonic flora, namely peritonitis or abdominal abscess [68,69].

This organism is a common cause of food poisoning due to the formation of the enterotoxin in the intestine. *C. perfringens* food poisoning is seldom fatal, being marked by diarrhea and nausea, with no vomiting and no fever [68,69].

Sources yielding *C. perfringens* include soil and marine sediment samples worldwide, clothing, raw milk, cheese, semi-preserved meat products, and venison. Like *E. coli*, *C. perfringens* does not multiply in most water environments and is a highly specific indicator of fecal pollution. Berzirtzoglou *et al.* [70] reported a comparative study on the occurrence of vegetative cells and spores of *Clostridium perfringens* in a polluted station of the lake Pamvotis, in rural North-West Greece. The numbers of *C. perfringens* varied according to the water depth. Sporulated forms were found in all sampling sites with the exception of the surface sampling.

#### 7.3.5. Lactobacillus

Lactobacilli are non-sporeforming Gram-positive long rods. There are more than thirty species in the genus. Most are microaerophillic, although some are obligate anaerobes. Cells are catalase-negative and obtain their energy by the fermentation of sugars, producing a variety of acids, alcohol and carbon dioxide. Lactobacilli have complex nutritional requirements and in agarized media may need the supplementation with aminoacids, peptides, fatty-acid esters, salts, nucleic acid derivatives and vitamins. Lactobacilli very rarely cause infections in humans [64].

#### 7.3.6. Enterococci

Enterococci are Gram-positive, non-sporeforming, catalase-negative ovoid cells. Cells occur singly, in pairs or short chains. Optimal growth for most species is 35–37 °C. Some will grow at 42–45 °C and at 10 °C. Growth requires complex nutrients but is usually abundant on commonly used bacteriological media. Cells are resistant to 40% bile, 0.4% azide, 6.5% sodium chloride, have β-glucosidase and hydrolyze esculin. The enterococci are facultative anaerobic but prefer anaerobic conditions [64,71].

The genus was separated from *Streptococcus* in the 1980s. Enterococci form relatively distinct groups. Members of such groups exhibit similar phenotypic characteristics and species delimitation can be difficult. The *E. faecalis* group contains, among others, *E. faecalis*. The *E. avium* group contains, among others, *E. avium*. The *E. faecium* group contains, among others, *E. faecium*, *E. durans* and *E. hirae*. The *E. gallinarum* group contains, among others, *E. gallinarum* [64,71].

Most species are part of the intestinal flora of mammals, reptiles, birds, and other animals. In the human digestive tract, *E. faecalis* is the prevailing species, although in particular situations, *E. faecium* may predominate. In poultry, *E. cecorum*, *E. durans*, *E. faecalis*, *E. faecium* and *E. hirae* and dominate the intestinal flora [64,71].

Enterococci have been increasingly isolated from a variety of nosocomial and other infections, mainly from the urinary tract and wound infections, bacteremias, and endocarditis [64,71].

Although enterococci are considered only a temporary part of the microflora of plants, in optimal conditions, cells can proliferate on their surfaces. *E. casseliflavus*, *E. faecalis*, *E. faecium*, *E. hirae*, *E. mundtii* and *E. sulfureus* have been isolated from plants. They are generally isolated more often from flowers than from buds or leaves [64,71].

Enterococci are naturally present in many kinds of foods, especially those of animal origin such as milk and milk products, meat and fermented sausages. Enterococci are usually considered secondary contaminants of food, although they often play a positive role in ripening and aroma development of some types of cheeses [64,71]. Although soil is not a natural habitat for enterococci, cells can be found in this habitat due to the transport by rain [64,71].

Environmental waters are not a natural habitat for enterococci and their presence in this milieu is considered the result of fecal pollution. The most common species found in environmental waters are *E. durans, E. faecalis*, *E. faecium* and *E. hirae*, and less commonly, *E. avium*, *E. cecorum*, *E. columbae* and *E. gallinarum*. However, pristine waters in Finland have been reported to contain *E. casseliflavus* [64,71].

In environmental samples (compost, sewage effluent, harbor sediments, brackish water and swimming pool water), Pinto *et al.* [72] reported the isolation of *E. casseliflavus*, *E. durans*, *E. faecalis*, *E. faecium*, *E. gallinarum* and *E. hirae. E. durans*, *E. faecium* and *E. hirae* were isolated from all sources except from harbor sediments. *E. raffinosus* was only isolated from compost and swimming pool water. *E. faecalis* and *E. faecium* accounted for the vast majority of enterococcal strains.

#### 7.3.7. Escherichia

*Escherichia*, a member of *Enterobacteriaceae*, are oxidase-negative catalase-positive straight rods that ferment lactose. Cells are positive in the Methyl-Red test, but negative in the Voges-Proskauer assay. Cells do not use citrate, do not produce H_2_S or lipase, and do not hydrolyze urea [73]. *E. coli* is a natural and essential part of the bacterial flora in the gut of humans and animals. Most *E. coli* strains are nonpathogenic and reside harmlessly in the colon. However, certain serotypes do play a role in intestinal and extra-intestinal diseases, such as urinary tract infections [43]. In a study of the enteric bacteria present in the feces of Australian mammals, Gordon and FitzGibbon [74] reported that *E. coli* was the commonest species, being isolated from nearly half of the species studied.

#### 7.3.8. Citrobacter

*Citrobacter*, a member of *Enterobacteriaceae*, are motile straight rods. Cells are oxidase-negative, catalase-positive and positive in the Methyl-Red test. Cells use citrate, are negative in the Voges-Proskauer test and do not decarboxylate lysine [73].

In a study of the enteric bacteria present in the feces of Australian mammals, Gordon and FitzGibbon [74] reported the isolation of *C. amalonaticus, C. freundii* and *C. koseri* (*C. diversus*). *Citrobacter* species can be isolated from different clinical sites. In particular, *C. freundii* is an intestinal inhabitant of humans that may sometimes have—or acquire—the ability to produce an enterotoxin and thus become an intestinal pathogen. *Citrobacter* is reported to occur in environments such as water, sewage, soil and food [75,76].

#### 7.3.9. Klebsiella and Raoultella

*Klebsiella* and *Raoultella* are *Enterobacteriaceae*, oxidase-negative catalase-positive non-motile straight rods, surrounded by a capsule. Cells decarboxylate lysine, but are ornithine and arginine dihydrolase negative. Cells grow on KCN, do not produce H_2_S and ferment most carbohydrates [73].

In humans, *K. pneumoniae* is present as commensal in the nasopharynx and in the intestinal tract. *Klebsiella* spp. can cause human diseases, ranging from asymptomatic colonization of the intestinal, urinary, or respiratory tract to fatal septicemia. *Klebsiella* are mostly considered nosocomial pathogens. *K. pneumoniae* and *Enterobacter aerogenes* (*K. mobilis*) are most frequently involved, although *K. oxytoca* and *R. planticola*, and rarely *R. terrigena*, can be found. In the hospital, the principal reservoir of *K. pneumoniae* is the gastrointestinal tract of patients. The principal vectors are the hands of personnel [77,78]. In a study of the enteric bacteria present in the feces of Australian mammals, Gordon and FitzGibbon [74] reported the isolation of *K. pneumoniae* and *K. oxytoca*.

Klebsiellae are ubitiquous in the environment. They have been found in a variety of environmental situations, such as soil, vegetation, or water, and they influence many biochemical and geochemical processes. They have been recovered from aquatic environments receiving industrial wastewaters, plant products, fresh vegetables, food with a high content of sugars and acids, frozen orange juice concentrate, sugarcane wastes, living trees, and plants and plant byproducts. They are commonly associated with wood, sawdust, and waters receiving industrial effluents from pulp and paper mills and textile finishing plants (see below). *Klebsiella* have been isolated from the root surfaces of various plants. *K. pneumoniae*, *K. oxytoca*, and *R. planticola* are all capable of fixing dinitrogen [77,78].

#### 7.3.10. Enterobacter

*Enterobacter* a member of *Enterobacteriaceae*, are motile straight rods. Cells are positive in the Voges-Proskauer test VP and in Simmons citrate agar. Cells do not decarboxylate lysine, but are ornithine positive. Malonate is usually utilized and gelatin is slowly liquefied. Cells do not produce H_2_S, deoxyribonuclease and lipase [73].

In a study of the enteric bacteria present in the feces of Australian mammals, Gordon and FitzGibbon [74] reported the isolation of *Enterobacter cloacae* subsp. *cloacae* (*E. cloacae*), *E. cancerogenus* (*E. taylorae*) and *E. aerogenes* (*Klebsiella mobilis*).

Before the widespread use of antibiotics, *Enterobacter* species were rarely found as pathogens, but these organisms are now increasingly encountered, causing nosocomial infections such as urinary tract infections and bacteremia. In addition, they occasionally cause community-acquired infections [79,80].

In the USA, the Surveillance and Control of Pathogens of Epidemiological Importance project analyzed 24,179 nosocomial bloodstream infections, from 1995–2002. *Enterobacter* species were the second most common gram-negative organism, behind *Pseudomonas aeruginosa*. Both bacteria were reported to each represent 4.7% of bloodstream infections in intensive care units. *Enterobacter* species represented 3.1% of bloodstream infections in non-intensive care units. Of nearly 75,000 gram-negative organisms collected from intensive care units’ patients in the USA, between 1993 and 2004, *Enterobacter* species comprised 13.5% of the isolates. Multidrug resistance increased over time, especially in infections caused by *E. cloacae* [81].

In the USA, the National Healthcare Safety Network reported a study on healthcare-associated infections between 2006 and 2007. They found *Enterobacter* species to be the eighth most common cause of healthcare-associated infections (5% of all infections) and the fourth most common gram-negative cause of these infections [82].

*Enterobacter cloacae* subsp. *cloacae* (*E. cloacae*) occurs in the intestinal tracts of humans and animals, in hospital environments, the skin, in water, sewage, soil, meat. Nitrogen-fixing strains have been isolated from the roots of rice plants. *E. amnigenus* has been mostly isolated from water, but some strains were isolated from clinical specimens from the respiratory tract, wounds and feces. *E. asburiae* strains were isolated from clinical specimens, mostly urine, respiratory tract, feces, wounds, and blood [79,80].

### 7.4. Origin of the Use of Fecal Indicator Bacteria

Historically, the design and use of indicators of fecal pollution comes from the end of the 19th to beginning of the 20th century. In 1880, von Fritsch described *Klebsiella pneumoniae* and *K. pneumoniae* subsp. *rhinoscleromatis* (*Klebsiella rhinoscleromatis*) as micro-organisms characteristically found in human feces [83]. In 1885, Escherich described several microorganisms in the feces of newborn and suckling babies. This included a motile, rod-shaped microorganism that caused milk to clot, which was named “*Bacterium coli commune*”. He observed that within a few weeks after birth, this bacterium became the dominant organism in the infant colon [6]. Also in 1885, Percy and Grace Frankland started the first routine bacteriological examination of water in London, using Robert Koch’s solid gelatin media to count bacteria [83]. In 1891, Percy and Grace Frankland came up with the concept that organisms characteristic of sewage must be identified to provide evidence of potentially dangerous pollution [83]. In 1892, Schardinger proposed that since “*Bacterium coli*” was a characteristic component of the fecal flora, its presence in water could be taken as an indication of the presence of fecal pollution and therefore of the potential presence of enteric pathogens [6]. Soon after the description of “*Bacterium coli*”, other bacteria were isolated from stools and water—*Klebsiella* in 1882 and *Enterobacter* in 1890 [6]. By 1893, the “Wurtz method” of enumerating “*Bacterium coli*”, by direct plating water samples on litmus lactose agar, was being used by sanitary bacteriologists. This was based on the concept of acid and gas production (detected by the Durham tube) from lactose as a diagnostic feature [6]. In 1905, MacConkey described his now famous MacConkey’s broth, which was diagnostic for lactose-fermenting bacteria tolerant of bile salts. Coliforms were already considered to be a heterogeneous group of organisms, many of which were not of fecal origin. The origins of the critical observation that “*Bacterium coli*” was largely fecal in origin while other coliforms were not, could be claimed by Winslow and Walker in 1907 [83].

Various classification schemes for coliforms have emerged. The earliest were those of MacConkey in 1909, which recognized 128 different coliform types, while Bergey and Deehan in 1908, identified 256. By the early 1920s, differentiation of coliforms had come to a series of correlations that suggested that indole production, gelatin liquefaction, sucrose fermentation and the Voges–Proskauer reaction were among the more important tests for determining fecal contamination. These developments culminated in the IMViC (Indole, Methyl Red, Voges–Proskauer and Citrate) tests for the differentiation of so-called fecal coliforms, soil coliforms and intermediates [83].

### 7.5. Fecal Indicator Bacteria

#### 7.5.1. Coliforms

Total coliforms are Gram-negative, oxidase-negative, non-sporeforming rods, that ferment lactose with gas production at 35–37 °C, after 48h, in a medium with bile salts and detergents [1,4,6,57,84]. When the test of coliforms is carried out with environmental waters, several species of the four *Enterobacteriaceae* genera *Escherichia*, *Klebsiella*, *Enterobacter* and *Citrobacter* give positive results and therefore are coliforms according to this definition. However, the environmental significance of these four genera is very disparate as discussed in the present text. Therefore, total coliform counts are not necessarily a measure of fecal pollution and indeed can have no relation with this cause [1,4,6,84].

Fecal coliforms (or thermotolerant coliforms) are traditionally defined as coliforms that ferment lactose at 44.5 °C in a medium with bile salts [1,4,57,84]. The range of species detected by the experimental procedure is much lower than that of total coliforms. With environmental polluted waters, only *E. coli*, and *K. oxytoca* and *K. pneumoniae* gave positive results in the test [85].

Traditional tests for total and fecal coliforms are carried out either by the multiple-tube fermentation technique or by filtration through membrane. The multiple-tube fermentation technique is used for medium or highly contaminated waters, and the filtration through membrane for low or very low contaminated waters. Filtration through membrane is a very sensitive technique since can detect one (culturable) cell in 500 or even 1,000 mL of water. However, both methods take several days to complete and do not detect viable but non-culturable bacteria [3,57,86]. These limitations stimulate the discovery of alternative methods, faster and, if possible, less prone to false negative results such as those caused by the viable but non-culturable bacteria.

The detection of β-D-galactosidase activity (at 37 °C) is usually a good marker for total coliforms in environmental waters, since most of these bacteria display this enzymatic activity [1,3,57,87–91]. Most *Escherichia*, *Citrobacter*, *Enterobacter*, *Klebsiella* and *Raoultella* strains have galactosidase. *Hafnia*, *Serratia* and *Yersinia* also possess this enzymatic activity. Most *Proteus*, *Salmonella* and *Edwardsiella* strains do not display β-galactosidase [92–95]. Ca. 10% of the coliform strains isolated from the environment do not have an active formic hydrogenolyase (cleaves formate with the formation of CO_2_) and therefore do not produce gas being undetected by the traditional techniques but are detected by the assay of β-galactosidase activity [57,96,97].

β-galactosidase cleaves lactose in glucose and galactose, and can be detected by using colored or fluorescent markers that change color after enzyme action, such as XGAL (5-bromo-4-chloro-3-indol-β-galactopyranoside) and ONPG (O-nitrophenyl-β-D-galactopyranoside) or MUGAL (4-methylumbelliferyl-β-D-galactopyranoside), respectively [57,96,97].

In environmental waters, the presence of *Aeromonas* or *Vibrio cholerae* can be a source of false positives in the β-D-galactosidase assay, since these bacteria have galactosidase, but are not coliforms [93,95,96,98]. Additionally, in particular environments, such as estuaries, β-galactosidase activity can overestimate total coliform count due to UV-stimulated enzymatic activity in certain bacteria such as *E. coli* [86].

The detection of β-D-glucuronidase activity (at 44.5 °C) is, generally, a good marker for fecal coliforms in environmental polluted waters and very specific for *E. coli* [13,85,87–91,97,99–101]. In Gram-negative bacteria, this enzymatic activity if found in most *E. coli* strains and in some *Salmonella* and *Shigella* strains [92–95,97,102]. *Aeromonas*, *Citrobacter*, *Enterobacter*, non-*coli Escherichia*, *Hafnia*, *Klebsiella*, *Proteus*, *Serratia*, *Vibrio*, *Yersinia*, and most *Salmonella* strains do not display β-glucuronidase activity [93–95,101,102].

β-D-glucuronidase activity can be detected by using colored or fluorescent markers that change color after enzyme action, such as XGLUC (5-bromo-4-chloro-3-indoxyl-β-d-glucuronide), IBDG (indoxil-β-glucuronide), and MUGLU (4-methylumbelliferyl-β-d-glucuronide), respectively [57,97,100].

The presence of this enzyme in some strains of *Bacteroides*, *Flavobacterium*, *Staphylococcus*, *Streptococcus*, in anaerobic corynebacteria and *Clostridium*, has also been reported [93,95–97,102]. β-D-glucuronidase activity in fecal bacteria other then *E. coli* (*Bacteroides*, bifidobacteria, clostridia, enterococci and *Lactobacillus*) is very limited [61]. Although all these glucuronidase positive bacteria could lead to false positive detections in the fecal coliform test, experimental results for environmental polluted waters indicate a significant correlation between fecal coliform detection using conventional techniques and the glucuronidase assay, suggesting that false positives are not significant [85,96].

The detection of total coliforms and fecal coliforms by enzymatic methods are much less time consuming than traditional techniques. With fluorescent markers and the use of a spectrofluorimeter the detection of coliforms can be performed in minutes [57,101]. However, in very low contaminated waters, enzymatic methods might not be able to detect coliform cells. Moreover, on-line monitoring of glucuronidase activity is currently too insensitive to replace culture based detection of *E. coli*. Nevertheless, on-line enzymatic methods can be a valuable complementary tool for high temporal resolution monitoring. More research is needed in order to enhance sensitive and lower detection limits of available on-line glucuronidase techniques.

The seminal work of Leclerc *et al.* [103] clarified the diversified roles that coliforms have in the environment and the real meanings of the tests on total coliforms and fecal coliforms. It was shown that *Enterobacteriaceae* encompass three groups of bacteria with very different roles in the environment. Group I harbored only *E. coli*. Since this species usually do not survive for long periods outside this environment (but see topic 10), it was considered a good and reliable indicator of fecal pollution (both animal and human). Group II, the “ubiquitary” group, encompassed several species of *Klebsiella* (*K. pneumoniae* and *K. oxytoca*), *Enterobacter* (*Enterobacter cloacae* subsp. *cloacae*, *E. aerogenes*) and *Citrobacter* (*C. amalonaticus*, *C. koseri* and *C. freundii*). These bacteria live in the animal and human gut, but also in the environment, and are easily isolated from the soil, polluted water and plants. Their presence in polluted waters does not necessarily indicate fecal pollution. Finally Group III was composed of *Raoultella planticola*, *R. terrigena, Enterobacter amnigenus* and *Kluyvera intermedia* (*Enterobacter intermedius*), *Serratia fonticola*, and the genera *Budvicia*, *Buttiauxella*, *Leclercia*, *Rahnella*, *Yersinia*, and most species of *Erwinia* and *Pantoea*. These bacteria live in fresh waters, plants and small animals. They grow at 4 °C, but not at 41 °C. They are not indicators of fecal pollution, although can be detected in the total coliform test. Leclerc *et al.* concluded that: (1) in the enterobacteria, *E. coli* is the only true and reliable indicator of fecal pollution in environmental waters; (2) the traditional total coliform test should be abandoned, since it can detect bacteria that have no connection with fecal pollution; (3) the detection of fecal coliforms must be carried out at 44.5 °C, and positive results confirmed by identification to species levels in order to exclude false positives such as *K. pneumoniae*.

#### 7.5.2. Streptococci and Enterococci

Fecal streptococci also belong to the traditional indicators of fecal pollution. Fecal streptococci are Gram-positive, catalase-negative, non-sporeforming cocci that grow at 35 °C in a medium containing bile salts and sodium azide. Cells hydrolyze esculin [1,4,57]. Azide is a strong inhibitor of the respiratory chain. Since streptococci are one of the very few bacteria that have no respiratory chain, the test is very specific for this group, and false positives are rarely found [104,105].

Fecal enterococci (*E. faecalis*, *E. faecium, E. avium* and *E. gallinarum*) are fecal streptococci that grow in the presence of 6.5% NaCl at 45 °C. Selective media use these particular characteristics in order to separate enterococci from the other streptococci [104,105].

Several studies [104,106] have reported on the microbiological composition of human and animal (cattle, chicken, deer, dog, fowl, goose, and swine) feces. *E. faecalis* and *E. faecium* were present in human and animal feces. However, whereas human feces almost have only these two enterococci, in the animals others species co-occur, like *E. avium*, *E. cecorum*, *E. durans*, *E. gallinarum* and *E. hirae*. It was concluded that in urban areas where contamination with dog and chicken feces is not likely, the best marker for human fecal pollution was *E. faecalis*.

The intestinal enterococci group has been used as an index of fecal pollution. In human feces, the numbers of intestinal enterococci are generally about an order of magnitude lower than those of *E. coli* (Table 6). Most species do not grow in environmental waters. In this milieu, fecal enterococci are able to survive longer, are more resistant to drying and chlorination, than *E. coli* [1,84].

However, caution should be taken with interpreting the results obtained by the enterococci procedure in water analysis. Enterococci and other group D-streptococci are present in many foods, especially those of animal origin. The isolation of *E. faecalis* and *E. faecium* was used to indicate fecal contamination of food. However, enterococci are now also considered as normal parts of the food microflora and not only as indicators for poor hygiene [104]. In addition, agricultural soils and crops with added with manure also harbor enterococci [105].

#### 7.5.3. The use of ratios between indicator counts

The ratio of counts of fecal coliforms to fecal streptococci has been proposed as a means to differentiating between contamination from human and animal sources. Ratios greater than 4 have been suggested to indicate a human source whereas ratios less than 0.7 suggest an animal source. This results from the fact that streptococcal concentrations in human feces are generally less than coliforms (Table 6). In contrast, in animal feces fecal streptococci generally outnumber fecal coliforms (Table 7). In urban sewage, fecal streptococci tend to be present in concentrations 10–100 times less than fecal coliforms [65].

Geldreich [107] summarized the information available on the fecal coliforms to fecal streptococci ratios in the feces of warmblooded animals, and reported the following values: human feces, 4.3; cattle, sheep, and poultry, from 0.104 to 0.421; and wild animals (including rabbits, field mice, chipmunks, and birds), 0.0008 to 0.043. Fecal coliforms to fecal streptococci ratios for the feces of wild animals appear to be at least 10-fold lower than those of domestic livestock.

Doran and Linn [108] reported a study of the runoff from a cow-calf pasture in eastern Nebraska (USA), monitored during a three-year period. It was concluded that the fecal coliforms to fecal streptococci ratio in pasture runoff was useful in identifying the relative contributions of cattle and wildlife and in evaluating the effects of cattle management and distribution on runoff water quality. Ratios below 0.05 were indicative of wildlife sources and ratios above 0.1 were characteristic of grazing cattle. Fecal coliforms to fecal streptococci ratios of diluted cattle waste in excess of 1 were interpreted as the result of differential aftergrowth and die-off between fecal coliforms and fecal streptococci. Ratios between 0.7 and 4.0 may indicate situations where cattle are localized close to sampling or outflow points.

However, the interpretation of this ratio should be cautious. It has been observed a shift in the ratio with time and distance from the fecal pollution source. This resulted from the fact that both in surface and groundwaters, fecal streptococci are more persistent than fecal coliforms. Therefore increasing the distance from the pollution point and with passing time, the ratio tends to decrease without a change in the nature of the pollution source [65]. For these reasons, this ratio has been considered by some authors as too unreliable to be useful in characterizing pollution sources [65,84].

The ratio of fecal enterococci to fecal streptococci differs among vertebrate species. Humans have a predominance of enterococci, whereas animals contain appreciable amounts of streptococci. However, since enterococci are also present in animals and are more persistent in the environment than other fecal streptococci, the identification of the enterococci and streptococci species present in polluted waters, and the concomitant calculation of this ratio is generally considered unreliable as an indicator of the source of fecal pollution [65].

#### 7.5.4. Limitations of coliform and enterococcus counts as indicator of fecal pollution

An extreme case of uselessness of the determination of total and fecal coliforms and enterococci in the assessment of fecal pollution has been demonstrated by several authors studying the microbiology of pulp and paper mill effluents.

Caplenas and Kanarek [109] reported a study of pulp and paper mills located in Wisconsin (USA). Fresh water supplies, re-cycled water within mills, treated effluent wastewater and waters receiving effluent wastes downstream, were assessed for the presence of fecal coliforms and *Klebsiella*. Wastewaters prior to treatment contained fecal coliforms and *Klebsiella*. Up to 84% of the fecal coliforms (detected by the standard test procedure) were indeed *Klebsiella*. In treated effluent wastewaters this value reached 90%. Treatment of the wastewater lowered the concentration of “true” fecal bacterial contamination, but since *Klebsiella* grew rapidly in the wastewaters, fecal coliform counts were high, although no true fecal contamination was involved. The source of *Klebsiella* was traced to the early pulping stages in the mills. *Klebsiella* maintains a wood, bark or soil reservoir. It was concluded that: (1) *Klebsiella* are ubiquitous in the pulp and paper mill industry processing stages; (2) the standard procedure for fecal coliform estimation is useless to assess the microbiological quality of the effluents of these industries; (3) The assay of *E. coli* should replace the fecal coliform detection procedure.

Gauthier *et al.* [110] and Gauthier and Archibald [58] reported two studies of seven pulp and paper mills in Ontario and Quebec, Canada. Total and fecal coliforms and enterococci were detected in nearly all the biotreatment, biosolids (sludges), and in-mill water system samples. In the mill samples, the majority of the fecal coliforms (detected by the standard test procedure) were *K. pneumoniae*, *Raoultella terrigena* and *Raoultella planticola*, with *E. coli* in minority. *E. faecalis* and *E. faecium* were detected in relatively large numbers in most samples from all of the seven mills examined. Other coliforms such as *Enterobacter* spp. and *Citrobacter freundii* were occasionally recovered from total and fecal coliform tubes. Biofilms established in the piping, tanks, and machinery where thermal and pH conditions permit were the most likely source of these bacteria. Analyses using two independent *Salmonella* detection/enumeration methods showed no detectable *Salmonella* cells in the sludges and final effluents of the five mills tested. It was concluded that for these particular systems, the determination of total and fecal coliforms and enterococci is useless and have no relationship with real fecal pollution. These studies also demonstrated the importance of checking the identities of bacteria causing the positive results in the tests. Both *Escherichia* and *Klebsiella* can give positive results in the fecal coliform test, but their ecological meaning is opposite.

Another important case of failure of the use of coliforms to detect fecal pollution was the 1993 *Cryptosporidium* outbreak in Milwaukee (USA).

*Cryptosporidium parvum*, a protozoan parasite that causes gastrointestinal illness, is transmitted by ingestion of oocysts excreted in human or animal feces. Typical modes of transmission include person to person, animal to person, by exposure to contaminated food or water [111,112].

From 1990 to 2000, at least 10 cryptosporidiosis outbreaks associated with contaminated drinking water were reported in the USA. In 1993, an estimated 403,000 residents of the greater Milwaukee area (Wisconsin, population, ca. 1.61 million) became ill when an ineffective filtration process led to the inadequate removal of *Cryptosporidium* oocysts in one of two municipal water-treatment plants [111,112].

It was the largest waterborne disease outbreak in documented USA history. Over the span of approximately two weeks, people became ill with stomach cramps, fever, diarrhea and dehydration caused by the pathogen. More than half the people who received residential drinking water from the southern water-treatment plant became ill, which was twice the rate of illness among people whose residential drinking water came mainly from the northern water-treatment plant. Over 54 deaths were attributed to this outbreak, mostly among the elderly and immunocompromised people, such as AIDS patients [111,112].

Standard microbiological water analysis was ineffective in detecting this parasite. Indeed, throughout the period from February to April, samples of treated water from both plants were negative for coliforms. The origin of the contamination was determined as water from Lake Michigan. No specific source of the *Cryptosporidium* was ever identified but runoff from abnormally heavy spring rains most likely carried the parasite to the lake [111,112].

#### 7.5.5. Clostridium perfringens

Sulphite-reducing clostridia, namely *Clostridium perfringens*, are spore-forming Gram-positive, non-motile, anaerobic, sulfite-reducing rods. *C. perfringens* is present in higher numbers in the feces of some animals, such as dogs, than in the feces of humans and less often in the feces of many other warm-blooded animals. The numbers excreted in feces are normally substantially lower than those of *E. coli.*

*Clostridium* spores are exceptionally resistant to unfavorable conditions in water environments, including UV irradiation, temperature and pH extremes, and disinfection processes, such as chlorination. Although clostridia probably do not growth in surface waters, the high resistance of their spores makes their presence ubiquitous in environmental waters [1,4,84,113].

The presence of chlorine in water rapidly inactivates indicator bacteria such as *E. coli* and coliforms, but it leaves the most resistant pathogens almost unaffected for several hours. This creates a false sense of security by providing negative coliform and negative *E. coli* results to authorities responsible for water testing. *Giardia* cysts, *Crystosporidium* oocysts, and human enteric viruses all have higher resistance to disinfectants and constitute a major public health risk if distribution system integrity is breached. *C. perfringens* spores are less affected by the residual concentrations of chlorine. Testing for the spores of this bacterium can probably provide an added margin of safety in the evaluation of treatment [114].

#### 7.5.6. Correlations between parameters used to assess fecal pollution

In environmental waters, several studies have reported significant correlations between indicators of fecal pollution and between indicators and pathogenic gastrointestinal bacteria.

Charriere *et al.* [115] reported a study of deep aquifer waters (raw waters and piped chlorinated waters) in Normandy, France. In heavily contaminated raw waters and in slightly contaminated treated waters, fecal coliforms and enterococci were correlated.

Martins *et al.* [116] reported a study of 60 public outdoor swimming pools in Sao Paulo city, Brazil. Total coliforms, fecal coliforms and fecal streptococci levels increased with number of bathers and water temperature, and decreased with chlorine levels. All these indicators were significantly correlated with each other.

Ferguson *et al.* [117] reported a study carried out in Georges River, in the Sydney region, Australia. In the water column, concentrations of fecal coliforms, fecal streptococci, *C. perfringens* spores were all positively correlated with each other. Isolation of *Salmonella* spp. were most frequent during rainfall and sewage overflow events. In the water column, 55% of samples contained *Salmonella* when fecal coliform densities exceeded 2,000 CFU/100 mL.

Medema *et al.* [118] reported a study of seven different fresh water sites normally used for triathlon competitions. Sites were small rivers, channels, lakes and harbors, and were influenced by sewage effluents and agricultural run-off. When data from all triathlons were pooled, geometric mean densities of fecal coliforms and *E. coli*, of *E. coli* and fecal enterococci and of fecal coliforms and fecal enterococci, were significantly correlated.

Polo *et al.* [119] reported a study of water samples obtained from 213 beaches, eight rivers and 14 freshwaters in north-eastern Spain. In freshwaters and heavily contaminated seawaters, *Salmonella* and fecal coliforms were correlated, while in less contaminated seawaters, the highest correlation was with between *Salmonella* and fecal streptococci.

Byamukama *et al.* [99] reported a study of the microbiology of Nakivubo channel, Uganda. This channel receives raw sewage from slums, industrial effluents, and discharges from a sewage treatment plant and from a complex of slaughterhouses. Water from eight sampling sites was assessed for the presence of total and fecal coliforms, *E. coli* and sulphite-reducing clostridia. All microbiological parameters were significantly correlated.

Noble *et al.* [120] reported a comparative determination of total coliforms, fecal coliforms and enterococci in 108 sites along the southern California coastline, USA. Results by traditional and enzymatic methods and from all three parameters were correlated.

Harwood *et al.* [121] reported a study on indicator and pathogenic microorganisms carried out in six wastewater reclamation facilities in the USA. Data from disinfected effluent (reclaimed water) samples were analyzed separately (by facility) and as a pooled data set (all facilities). Significant correlations between indicator organism concentrations were observed in the pooled data sets, namely for total and fecal coliforms.

Cabral and Marques [85] reported a microbiological study of a polluted river (Febros) in the Great Oporto area, northwest Portugal. Total and fecal coliforms, fecal streptococci and enterococci were all significantly correlated with each other.

Touron *et al.* [122] reported a study carried out in the Seine estuary, France. Water was sampled at nine stations (along an upstream/downstream transect of 156 km), during nine years, for fecal coliforms, *E. coli*, enterococci and *Clostridium perfringens* spores. At the upstream part of the estuary (at Poses), *Salmonella* and fecal coliforms, and *E. coli* and enterococci counts were correlated. At the mouth of the estuary (at Honfleur), significant correlation was found for *Salmonella* and enterococci counts. No significant correlation between concentrations of any combination of indicator organism and pathogen was observed.

Wilkes *et al.* [59] reported a comparative study on the presence and concentration of several pathogenic and indicator bacteria in the surface water of a Canadian river. Surface water was collected within the South Nation River basin in eastern Ontario, from the river proper, and from several lower stream order tributaries. Using data aggregated during the entire multi-year study, significant correlations were found among all indicator bacteria - total and fecal coliforms, *E. coli*, *Enterococcus*, and *C. perfringens*.

However, in others studies no correlation was found between the different fecal indicator bacteria. Garrido-Pérez *et al.* [123] reported a study of the bathing seawater quality in 18 Spanish beaches near the Strait of Gibraltar. Sample locations were selected as a single point located in the area of highest bather density of each beach. No significant correlation was found between fecal coliforms and *Clostridium perfringens* counts in the bathing seawater.

## 8. Fecal Indicator Chemical Compounds

Several chemical substances have been used as markers of fecal pollution in environmental waters. Caffeine is present in several beverages and in many pharmaceutical products. It is excreted in the urine of individuals who have ingested the substance. The main source of caffeine in domestic wastewaters is excretion following consumption of coffee, tea, soft drinks, or medication. Levels of caffeine in domestic wastewater have been measured to be between 20 and 300 μg/L. Levels in receiving waters are much lower due to significant dilution. Due to its high solubility, low octanol-water partition coefficient, insignificant volatility and clear anthropogenic origin, the presence of caffeine in environmental waters can be a good marker for human fecal pollution [124–129].

However, relationships between fecal indicator bacteria and caffeine are variable. Wu *et al.* [129] reported a study carried out in the Rochor Canal and Marina Bay, Singapore. In Rochor Canal, the highest concentration of caffeine (1.35 ng/mL) was found at downstream, and the lowest (0.68 and 0.37 ng/mL) were determined at middle and upstream points. At Marina Bay, the concentration of caffeine was in the range of 0.41–0.96 ng/mL. Fecal coliform concentrations were very high, exceeding 5,000 CFU/100 mL. Caffeine and fecal coliform concentrations were significantly correlated in Rochor Canal samples, but no significant correlation was observed in Martina Bay water samples.

The use of caffeine as marker of fecal pollution has additional important limitations. Caffeine is often present in the urban environment from numerous plant species debris as well as from human “dumping” of coffee wastes. In addition, the current analytical methods used are relatively complex and expensive [124].

Coprostanol (5β-cholestan-3β-ol) is a fecal stanol that is formed by indigenous bacteria present in the gut of humans and higher animals, during catabolism of cholesterol. It is the main stanol present in human feces (24 to 89% of total steroids) and in domestic wastewater. Based on these facts, it has been proposed as a chemical indicator of human fecal pollution. Feces from pigs and cats also contain coprostanol, but at much lower levels. Additional fecal stanols, such as 24-ethylcoprostanol, were found to be predominant in herbivores, such as cows, horses, and sheep, suggesting potential use of this chemical as an indicator of fecal pollution from these sources. Reported half-lives of coprostanol in aerobic conditions are generally lower than 10 days at 20 °C. Thus, the presence of coprostanol in an aerobic environment can be considered an indication of recent fecal input to the waters. [124,128,130].

Isobe *et al.* [130] reported a study carried out in the Mekong Delta (Vietnam) and in the Tokyo metropolitan area. During the wet season in the Mekong Delta, higher bacterial densities were observed in rivers, probably due to the higher bacterial inputs from soil particles with runoff. In Tokyo, higher bacterial densities were usually observed during summer, followed by those in the typhoon aftermath and winter. Significant correlations between the concentrations of *E. coli* and coprostanol (log scale) were found in all surveys. It was concluded that the determination of coprostanol can improve standard microbiological assays of fecal pollution.

Reports from several regions throughout the world indicate variable quantitative relationships between fecal coliform densities and coprostanol concentrations. In the Derwent Estuary and Sydney region (Australia), coprostanol concentration of 400 ng/L corresponded to 1,000 CFU of fecal coliforms/100 mL. In the Mekong Delta, during the wet season, this coliform density corresponded to 30 ng coprostanol/L, and in the dry seasons, 100 ng/L. In Tokyo metropolitan area, these values were 30 ng coprostanol/L, in summer, and 100 ng coprostanol/L, in a typhoon aftermath. These differences were interpreted as a result of differences in water temperature and soil particle concentration [130].

Fecal sterol analysis, although expensive and complex, has resolved problems of source attribution in urban and rural environments not possible with use of traditional fecal indicator bacteria [124]. These chemical indicators are especially useful in environments which allow survival and growth of fecal bacteria. For instance, in tropical regions, characterized by high temperatures and frequent rainstorms that facilitate erosion of soils, fecal bacteria can proliferate in environmental waters reaching densities that are not representative of real sewage inputs in the environment [130].

## 9. Sources of Fecal Bacterial Pollution of Environmental Waters

### 9.1. Sources of Surface and Groundwater Contamination

Determinations carried out in the sewage systems of urbanized areas have confirmed the presence of high numbers of intestinal bacteria. Treatment of sewage reduces the concentration of these bacteria by 1–2 logs, but effluent still contains high levels of intestinal bacteria (Table 8). Effluents from sewage treatment plants can be a source of contamination of surface waters with fecal bacteria.

Septic tanks, cesspools, latrines and other on-site systems are widely used for wastewater storage and treatment. The water percolating from these facilities contains bacteria that may contaminate groundwater supplies.

Many farmers use cellars, tanks or landfills to store manure. Water leaching from these storage sites may also contaminate groundwater, especially during periods of rainfall. The application of animal manure to agricultural lands as fertilizer is common practice throughout the world. Bacteria present in the manure may leach into the groundwater.

The potential for bacteria present in human and animal wastes to contaminate water in nearby wells needs special attention [131]. An important source of contamination of surface and ground waters is runoff water from agricultural and pasture lands, and urban areas.

Fecal bacteria enter surface water by direct deposit of feces and by overland runoff. The movement of animal wastes into surface waters can be a major factor contributing to the pollution of available water in many regions. Over one-third of the land area of USA is used for grazing livestock and receives 50% of all livestock wastes.

In a study reported by Doran and Linn [108], runoff from a cow-calf pasture in eastern Nebraska was monitored during a three-year period. Rainfall runoff from the grazed area contained 5 to 10 times more fecal coliforms than runoff from the fenced, ungrazed area. However, fecal streptococci counts were higher in runoff from the ungrazed area and reflected the contributions from wildlife.

Urban and suburban areas are dominated by impervious cover. During storms, rainwater flows across these impervious surfaces, mobilizing contaminants. The pollutants carried in runoff originate from a variety of urban and suburban nonpoint sources. Contaminants commonly found in stormwater runoff include fecal and pathogenic bacteria. Stormwater transports pollutants to water bodies such as lakes and streams [132].

Enterococci and *E. coli* can be found in high numbers in most storm drains and creeks. In Southern California (USA), Ferguson *et al.* [133] found high levels of enterococci (*Enterococcus faecalis*, *Enterococcus faecium*, *Enterococcus hirae*, *Enterococcus casseliflavus* and *Enterococcus mundtii*) in intertidal sediments in a seasonal river, and near a storm drain outlet.

### 9.2. Survival in Surface Water

Most intestinal bacteria that contaminate environmental waters are not able to survive and multiply in this environment. Survival rates vary widely among fecal bacteria introduced in environmental waters. Pathogenic enteric bacteria and *E. coli* display low survival rates (Table 9).

The ability of fecal bacteria to survive in environmental waters generally increases as the temperature decreases. Others factors that influence survival include dissolved organic carbon concentration, sunlight intensity and the ability to enter the viable but non-culturable state [131].

In a comparative study on the survival of 10 different coliform species (*E. coli*, *Citrobacter freundii*, *Citrobacter youngae*, *Klebsiella pneumoniae*, *K. oxytoca*, *Enterobacter amnigenus*, *Enterobacter cloacae* subsp. *cloacae*, and *Pantoea agglomerans* (*Enterobacter agglomerans*)) inoculated in sterilized river water with different concentrations of dissolved organic carbon, Boualam *et al.* [134] found that only *C. freundii*, *K. pneumoniae* and *E. cloacae* subsp. *cloacae* remained cultivable after 96 hours of incubation. In a posterior study, using the same bacteria and medium, Boualam *et al.* [135] found that after 28 days, only *C. freundii* and *E. cloacae* subsp. *cloacae* survived.

Baudišová [136] reported a comparative study on the survival of total coliforms, fecal coliforms and *E. coli*, in sterile and non-sterile river water. In sterile water, all bacteria survived for many months. However, in non-sterile conditions (closer to true environmental conditions), the elimination rate of all bacteria was considerably faster. Total coliforms survived the longest and *E. coli* the shortest.

### 9.3. Survival in Groundwater

Survival of bacteria in groundwater is influenced by several factors, namely the survival in soil, since in order to reach the groundwater bacteria have to percolate through the soil. Generally, survival in soil (and concomitantly in groundwater) is enhanced by low temperatures, high soil humidity, neutral or alkaline soil pH and the presence of organic carbon [131].

## 10. Which Indicators of Fecal Pollution Should be Used?

Several fecal indicator bacteria in environmental waters are in current use. From these stand out fecal coliforms, *E. coli* and enterococci [1,6,137]. In environmental waters, most fecal coliform strains are *E. coli*.

In particular situations, the presence of *E. coli* is definitively not associated with fecal pollution. These situations were firstly detected in some African countries, namely Nigeria, Ivory Coast and New Guinea (although not in others, such as Uganda) [42,99].

Recent studies carried out in temperate zones indicated that *E. coli* can persist in secondary, nonhost habitats, outside the hot tropical areas, and become naturalized in these habitats. Byappanahalli *et al.* [138] reported that *E. coli* could be isolated from coastal temperate forest soils in Indiana (USA). The aquatic alga *Cladophora glomerata* (L.) from several Lake Michigan beaches was shown to harbor high densities of *E. coli* [139].

Ishii *et al.* [140] reported a study on the survival of *E. coli* in temperate riverine soils of northern Minnesota (USA). Viable *E. coli* populations were repeatedly isolated from northern temperate soils in three Lake Superior watersheds. Seasonal variation in the population density of soilborne *E. coli* was observed; the greatest cell densities were found in the summer to fall, and the lowest numbers, occurred during the winter to spring months. Horizontal, fluorophore-enhanced repetitive extragenic palindromic PCR (HFERP) DNA fingerprint analyses indicated that identical soilborne *E. coli* genotypes, overwintered in frozen soil and were present over time, and that these strains were different from *E. coli* strains obtained from wildlife commonly found in the studied habitats or river water. Soilborne *E. coli* strains had HFERP DNA fingerprints that were unique to specific soils and locations. In laboratory studies, naturalized *E. coli* strains had the ability to grow and replicate to high cell densities, in nonsterile soils when incubated at 30 or 37 °C and survived longer than 1 month when soil temperatures were lower than 25 °C. It was concluded that these *E. coli* strains became naturalized, autochthonous members of the soil microbial community.

In a latter paper, Ksoll *et al.* [141] studied epilithic periphyton communities at three sites on the Minnesota shoreline of Lake Superior (USA). Fecal coliform densities increased up to 4 orders of magnitude in early summer, and decreased during autumn. HFERP DNA fingerprint analyses indicated that waterfowl (geese, terns, and gulls) were the major primary source of periphyton *E. coli* strains that could be identified. Periphyton and sewage effluent were also major potential sources. Several periphyton *E. coli* isolates were genotypically identical, repeatedly isolated over time. Inoculated *E. coli* rapidly colonized natural periphyton in laboratory microcosms and persisted for several weeks, and some cells were released to the overlying water. It was concluded that *E. coli* had became a naturalized member of the bacterial periphyton communities.

The presence, persistence, and possible naturalization of *E. coli* in these habitats can confound the use of fecal coliforms as a reliable indicator of recent fecal contamination of environmental waters. Future studies should consider other nonhost habitats as potential sources of fecal coliform bacteria in aquatic environments.

Considering these limitations, it appears to be advisable, in order to check the microbiological quality of drinking water, to complement the determination of *Escherichia coli* with the assay of enterococci. This rationale that has been followed, for many years, in the making of the drinking-water legislation in the European Union.

However, for many developing countries, where limited financial resources are the norm and reality, the routine determination of these two parameters can be difficult to implement. In these circumstances, it appears common sense that is better to determine a (good) parameter, such as *Escherichia coli*, than have no analysis done.

In this context, USA legislation emerges as a pragmatic approach to the problem. According to the American legislation, total coliforms are the routine parameter to be determined. Only when these determinations are repeatedly positive, it is mandatory to assess fecal coliforms [142,143]. Although total coliforms are not necessarily fecal bacteria, the rationale behind this system is correct, since: (1) a positive test in fecal coliforms (which is our target) is necessarily positive in the total coliform procedure; (2) the inverse is not necessarily true; (3) total coliforms are easily and cheaply assayed in waters.

As an alternative to the determination of both *E. coli* and enterococci, the assay of ammonia in environmental waters can be useful and complement the determination of fecal coliforms.

Ammonia is one of the key molecules in the nitrogen cycle. The presence of ammonia in surface waters can be due to direct contamination by agricultural fertilizers, and/or to microbial degradation of proteins, nucleic acids and urea, implying therefore the presence of a considerable concentration of organic matter in the water. Ammonia is rapidly oxidized in the environment and is typically found in natural waters at concentrations less than 0.1 mg/L. Concentrations significantly above this indicate gross contamination by fresh sanitary waste, where ammonia levels are typically very high (tens or hundreds of mg/L) [84].

Espigares *et al.* [144] reported a comparative study of chemical and microbiological indicators (total and fecal coliforms, fecal streptococci and sulphite-reducing clostridia) in a stretch of the Guadalquivir River (Spain) and its affluents. Total coliforms were correlated with fecal coliforms, but were not correlated with fecal streptococci and clostridia. Fecal coliforms were correlated with the other indicators. Fecal streptococci and sulphite-reducing clostridia were correlated with the other indicators except for total coliforms. All these microbiological indicators were correlated with dissolved oxygen (negatively), dissolved organic carbon and ammonia (positively). Cabral and Marques [85] in a study of a polluted river (Febros) in the Great Oporto area (northwest Portugal) found that ammonia was significantly correlated with all the microbiological assayed parameters—total and fecal coliforms, fecal streptococci and enterococci. These correlations are most probably due to the carry-over of organic matter in wastewaters, and to a high microbial ammonification activity [85].

Simple and rapid in-field tests and automated and continuous systems are available for the assay of ammonia in environmental waters. More studies are needed in order to confirm the use of ammonia as a reliable parameter in a preliminary screening for emergency fecal pollution outbreaks.

## 11. Conclusions

1. Safe drinking water for all is one of the major challenges of the 21st century.
2. Microbiological control of drinking water should be the norm everywhere.
3. Routine basic microbiological analysis of drinking water should be carried out by assaying the presence of *Escherichia coli* by the culture methods. On-line monitoring of glucuronidase activity is currently too insensitive to replace culture based detection of *E. coli* but is a valuable complementary tool for high temporal resolution monitoring. Whenever financial resources are available, coliform determinations should be complemented with the quantification of enterococci.
4. More studies are needed in order to check if ammonia is reliable for a preliminary screening for emergency fecal pollution outbreaks.
5. Financial resources should be devoted to a better understanding of the ecology and behavior of human and animal fecal bacteria in environmental waters.

## Figures and Tables

**Table 1.** The main bacterial diseases transmitted through drinking water.

**Disease**	**Causal bacterial agent**
Cholera	*Vibrio cholerae*, serovarieties O1 and O139
Gastroenteritis caused by vibrios	Mainly *Vibrio parahaemolyticus*
Typhoid fever and other serious salmonellosis	*Salmonella enterica* subsp. *enterica* serovar Paratyphi
	*Salmonella enterica* subsp. *enterica* serovar Typhi
	*Salmonella enterica* subsp. *enterica* serovar Typhimurium
Bacillary dysentery or shigellosis	*Shigella dysenteriae*
	*Shigella flexneri*
	*Shigella boydii*
	*Shigella sonnei*
Acute diarrheas and gastroenteritis	*Escherichia coli*, particularly serotypes such as O148, O157 and O124

**Table 2.** Main species of *Vibrio* and their occurrence in human clinical specimens^a^.

**Main species**	**Occurrence in human clinical specimens**	
	**Intestinal**	**Extra-intestinal**
*Vibrio alginolyticus*	+	++
*Vibrio cholerae* O1 and O139	+++++	+
*Vibrio cholerae* non O1 or O139	++	++
*Aliivibrio fischeri* (*Vibrio fischeri*)	−	−
*Vibrio fluvialis*	++	−
*Vibrio furnissii*	++	−
*Vibrio harveyi*	−	+
*Grimontia hollisae* (*Vibrio hollisae*)	++	−
*Vibrio mimicus*	++	+
*Vibrio natriegens*	−	−
*Vibrio parahaemolyticus*	++++	+
*Vibrio vulnificus*	+	+++

a Adapted from [7,8]. Nomenclature according to [9]. The symbols give the relative frequency of each organism in human clinical specimens, and apply to the whole World, rather than to a particular country.

**Table 3.** Subdivision of *Vibrio cholerae* below the species level^a^.

**Serovariety**	**Serotype**	**Biotype**
O1	Inaba	Classical
		El Tor
	Ogawa	Classical
		El Tor
	Hikojima	
O139		
others		

a Adapted from [8].

**Table 4.** Current taxonomy and nomenclature of the genus *Salmonella*. Habitat and pathogenicity of main serovars^a^.

**Species**	**Sub-species**	**Main serovars (from a total of ca. 1,443)**	**Habitat and pathogenicity**
*Salmonella enterica*	*Salmonella enterica* subsp. *enterica*	Abortusovis	Pathogenic to sheeps.
		Choleraesuis	Pathogenic to humans and animals.
		Enteritidis	Ubiquitous and frequently the cause of infections in humans and animals. Very frequent agent of gastroenteritis in humans.
		Gallinarum	Isolated chiefly from chickens and other birds. Causal agent of fowl thyphoid.
		Paratyphi A	Pathogenic only to humans. Causes paratyphoid fever.
		Paratyphi B	Causes paratyphoid fever in humans and very rarely infects animals.
		Paratyphi C	Causes paratyphoid fever in humans.
		Typhi	Pathogenic only to humans, causing typhoid fever.
			Transmitted by water and food contaminated with feces.
		Typhimurium	Ubiquitous and frequently the cause of infections in humans and animals. Very frequently, the causal agent of gastroenteritis in humans.
		Typhisuis	Pathogenic to swines.
	*Salmonella enterica* subsp. *arizonae*	At least 94 serovars.	
	*Salmonella enterica* subsp. *diarizonae*	At least 323 serovars.	
	*Salmonella enterica* subsp. *houtenae*	At least 70 serovars.	Isolated mainly from cold-blooded animals and from the environment. Not pathogenic to humans.
	*Salmonella enterica* subsp. *indica*	At least 11 serovars.	
	*Salmonella enterica* subsp. *salamae*	At least 488 serovars.	
*Salmonella bongori*		At least 20 serovars.	

a Adapted from [29]. Nomenclature according to [9].

**Table 5.** Current taxonomy and nomenclature of the genus *Shigella*. Habitat and pathogenicity of species^a^.

**Species**	**Main serotypes**	**Habitat and pathogenicity**
*Shigella dysenteriae*	15 serotypes.	Intestinal pathogens of humans and primates, causing bacillary dysentery.
*Shigella flexneri*	8 serotypes 9 subserotypes	Humans are the primary reservoir. A long-term carrier state occurs in few cases.
*Shigella boydii*	19 serotypes	*Shigella dysenteriae* serotype 1 causes more severe disease then other serotypes and produces a potent exotoxin (Shiga toxin). Large epidemics in developing countries are commonly caused by serotype 1. Diseases caused by other serotypes may be mild or severe.
*Shigella sonnei*	1 serotype	*Shigella sonnei* illness is usually milder than that caused by other *Shigella* species.

a Adapted from [34]. Nomenclature according to [9].

**Table 6.** Total viable count in feces of healthy humans (children, adults and elderly)^a^.

**Microbial group**	**Log**_**10**_**CFU/g feces**
*Bacteroides*	11.3*
*Eubacterium*	10.7*
*Bifidobacterium*	10.2*
*Ruminococcus*	10.2*
*Peptostreptococcus*	10.1*
*Peptococcus*	10.0*
*Clostridium*	9.8*
*Lactobacillus*	9.6*
*Propionobacterium*	9.4*
*Actinomyces*	9.2*
*Methanobrevibacter*	8.8*
*Desulphovibrio*	8.4*
*Fusobacterium*	8.4*
Enterococci	3.5–7.2**
*Enterobacteriaceae*	5.9–8.0**
*Escherichia coli*	7.5–7.7**
*Citrobacter*	3.3**
*Klebsiella*	2.4**
Yeasts	1.0–2.5**

a Adapted from [61–63]** and [64]*.

* Values expressed as dry weight.

** Values expressed as wet weight.

**Table 7.** Bacteria in the feces of farm and domestic warm-blooded animals^a^.

**Animal**	**Log**_**10**_**cells/g wet weight feces**		
	**Fecal coliforms**	**Fecal streptococci**	***Clostridium perfringens***
Chicken	5.4	6.1	2.3
Duck	7.5	7.7	-
Horse	4.1	6.8	< 0
Pig	6.5	7.9	3.6
Sheep	7.2	7.6	5.3
Turkey	5.5	6.4	-
Cat	6.9	7.4	7.4
Dog	7.1	9.0	8.4

a Adapted from [83].

**Table 8.** Typical concentrations of selected bacteria in raw and treated domestic wastewater^a^.

**Bacterial group**	**Raw sewage (cells/ml)**	**Treated effluent (cells/ml)**
*Salmonella*	10^−1^ – 10^1^	10^−1^ – 10^1^
Total coliforms	10^4^ – 10^6^	10^3^ – 10^5^
Fecal coliforms	10^3^ – 10^5^	10^2^ – 10^4^
Enterococci	10^3^ – 10^4^	10^1^ – 10^3^
*Clostridium perfringens*	10^2^ – 10^3^	10^1^ – 10^2^

a Adapted from Medema *et al.* [131].

**Table 9.** Reduction times for fecal bacteria in surface waters^a^.

**Bacterial group**	**Time for 50% reduction in concentration (days)**
Total coliforms	0.9
*E. coli*	1.5–3
Enterococci	0.9–4
*Clostridium perfringens*	60 – > 300
*Salmonella*	0.1–0.67
*Shigella*	1

a Adapted from Medema *et al.* [131].

**Table 10.** Disappearance rates of fecal bacteria in groundwaters^a^.

**Bacterial group**	**Disappearance rate (per day)**
*E. coli*	0.063–0.36
Fecal streptococci	0.03–0.24
*Clostridium bifermentans* spores	0.00
*Salmonella enterica* subsp*. enterica* serovar Typhimurium	0.13–0.22

a Adapted from Medema *et al.* [131].
